# ﻿*Thyridiumlauri* sp. nov. (Thyridiaceae, Thyridiales): a new pathogenic fungal species of bay laurel from Italy

**DOI:** 10.3897/mycokeys.110.129228

**Published:** 2024-11-15

**Authors:** Giuseppa Rosaria Leonardi, Dalia Aiello, Chiara Di Pietro, Antonio Gugliuzzo, Giovanna Tropea Garzia, Giancarlo Polizzi, Hermann Voglmayr

**Affiliations:** 1 Department of Agriculture, Food and Environment, University of Catania, Via Santa Sofia 100, 95123 Catania, Italy University of Catania Catania Italy; 2 Department of Botany and Biodiversity Research, University of Vienna, Rennweg 14, 1030 Vienna, Austria University of Vienna Vienna Austria

**Keywords:** Fungal diseases, *Laurusnobilis*, phylogeny, stem blight and internal necrosis, taxonomy, *Xylosandruscompactus*

## Abstract

*Laurusnobilis* is an important Mediterranean tree and shrub native to Italy that is also commercially grown as spice and ornamental plant. Field surveys conducted since 2021 in Sicily (Italy) revealed that bay laurel plants in urban and private gardens and nurseries were severely affected by symptoms of stem blight and internal necrosis, which were associated with ambrosia beetle entry holes in the bark and internal wood galleries. The occurring ambrosia beetle was identified as *Xylosandruscompactus*, an invasive wood-boring pest previously reported from Sicily. Investigation of fungi from symptomatic tissues primarily resulted in the isolation of *Thyridium*-like colonies. The main symbiont of *X.compactus*, *Ambrosiellaxylebori*, was also isolated from infested plants. Phylogenetic analyses of a combined matrix of ITS, LSU, *act1*, *rpb2*, *tef1*, and *tub2* gene regions revealed that the isolated *Thyridium*-like colonies represent a new fungal species within the genus *Thyridium*. Based on both phylogeny and morphology, the new isolated fungus is described as *Thyridiumlauri***sp. nov.** Moreover, two recently described species, *Phialemoniopsishipposidericola* and *Phialemoniopsisxishuangbannaensis*, are transferred to the genus *Thyridium* due to the confirmed synonymy of both genera, as supported by molecular phylogenies. Pathogenicity test conducted on potted plants demonstrated that *T.lauri* is pathogenic to bay laurel, causing internal necrosis and stem blight. The new species was consistently re-isolated from the symptomatic tissue beyond the inoculation point, thereby fulfilling Koch’s postulates. This study represents the first report of a new pathogenic fungus, *T.lauri*, causing stem blight and internal necrosis of bay laurel plants and associated with infestation of the invasive ambrosia beetle *X.compactus*.

## ﻿Introduction

Lauraceae is one of the largest families of trees and shrubs, distributed worldwide with approximately 55 genera and 3,000 species (Renner 2011; Zhang et al. 2023). This family includes economically important plants such as avocado (*Perseaamericana* Mill.), camphor (*Cinnamomumcamphora* (L.) Sieb.), cinnamon (*Cinnamomumzeylanicum* Blume), and bay laurel (*Laurusnobilis* L.). Bay laurel, also commonly known as sweet bay, is an evergreen tree or shrub naturally widespread across many countries of the Mediterranean basin, along the Atlantic coast of the Iberian Peninsula and the southern coast of the Black Sea (Giacobbe 1939; Rodríguez-Sánchez and Arroyo 2008). This plant is also cultivated in warm regions of the Americas, Eastern Asia, the Balkans, and Asia Minor (Batool et al. 2020; Singletary 2021). The economic importance of bay laurel lies in its use in public and private gardens as an ornamental tree or hedging plant (Raviv et al. 1982; Malaspina et al. 2022), as well as in its aromatic leaves, which serve as an important spice and flavoring agent in the culinary and food industries worldwide (Stefanaki and van Andel 2021). Traditionally, bay laurel leaves have been used in medicine by local people of Mediterranean countries for their biological properties, which are positively correlated with human health (Fang et al. 2005; Motti and de Falco 2021). Several recent studies have reported interest in the production of bay laurel plants due to the pharmacological and cosmetic properties of their essential oils and extracts (Lubbe and Verpoorte 2011; Ordoudi et al. 2022). Although bay laurel is investigated worldwide for its diverse potential uses, there is limited data available on fungal diseases affecting this plant. In Italy, leaf spots and stem blight caused by *Pestalotiopsisuvicola* (Speg.) Bissett, as well as crown and root rot caused by *Calonectriailicicola* Boedijn & Reitsma have been reported on young potted plants (Vitale and Polizzi 2005; Polizzi et al. 2012). Furthermore, fungal diseases transmitted by bark and ambrosia beetles may pose a significant limitation to the cultivation of bay laurel on a global scale. Fungi closely associated with bark and ambrosia beetles are capable of infecting both the bark and wood tissues, resulting in severe diseases in the host tree. Among the fungi carried by these beetles, a quarantine pathogen is *Harringtonialauricola* (T.C. Harr., Fraedrich & Aghayeva) Z.W. de Beer & M. Procter (formerly *Raffaelealauricola*) (Ascomycota, Ophiostomatales) which causes a lethal vascular disease known as laurel wilt. This disease affects numerous species in the Lauraceae family, including bay laurel (Mayfield et al. 2008; Hughes et al. 2014, 2017; Eaton et al. 2023). This fungus has rapidly spread across the United States vectored by several species of ambrosia beetles, particularly *Xyleborusglabratus* Eichhoff (Coleoptera: Curculionidae), commonly known as the redbay ambrosia beetle (Ploetz et al. 2017).

Fungal species of the genus *Geosmithia*, particularly *Geosmithialangdonii* M. Kolařík, Kubátová & Pažoutová, *G.pallida* (G. Sm.) M. Kolařík, Kubátová & Pažoutová, and *G.putterillii* (Thom) Pitt (Ascomycota, Hypocreales), have been reported to be vectored by *Liparthrumcolchicum* Semenov (Coleoptera: Curculionidae: Scolytinae), a phloem-feeding bark beetle, and are associated with dried twigs of bay laurel in France (Jordal et al. 2004; Kolarík et al. 2004, 2007) and Italy (Benvenuti et al. 2021; Vitale et al. 2021). *Ambrosiellaxylebori* Brader ex Arx & Hennebert (Ascomycota, Ceratocystidaceae), along with *Fusarium* spp. and *Bionectria* sp., were involved in necrosis of twigs and branches of bay laurel infested by the ambrosia beetle *Xylosandruscompactus* (Eichhoff) (Coleoptera: Curculionidae) (Vannini et al. 2017). This invasive wood-boring insect pest, native to subtropical and tropical Asia, has been introduced to various subtropical regions worldwide, with its first report in the Mediterranean region coming from Italy in 2011 (FITOLAB 2011; Francardi et al. 2017). Currently, *X.compactus* is regarded as an emerging pest affecting various trees and ornamental plants in Italy (Francardi et al. 2017; Vannini et al. 2017; Gugliuzzo et al. 2019a, 2019b; Contarini et al. 2020), and bay laurel is reported as one of the preferred host plants of this ambrosia beetle in the Mediterranean regions (Francardi et al. 2012; Barnouin et al. 2020; Riba-Flinch et al. 2021; Hızal et al. 2023). In Sicily, heavy infestations by *X.compactus* have primarily been reported on carob (*Ceratoniasiliqua* L.), with less severe occurrences on bay laurel (Gugliuzzo et al. 2019c, 2023a).

Since 2021, bay laurel plants showing infestations of wood-boring insects associated with stem blight and internal necrosis have been observed in different nurseries of Sicily during field samplings following disease detection by farmers. From 2022 to 2024, additional surveys and observations were conducted in collaboration with the Regional Phytosanitary Service (Sicily, Italy) in street and park shrubs, private gardens, and nurseries with mother plants for tree propagation located in the Catania and Syracuse provinces. In most cases, the survey conduction revealed stem blight and internal necrosis, and the decline or death of numerous bay laurel trees, consistently accompanied by insect entry holes in the bark and internal galleries. As many fungal species have been reported worldwide for causing stem blight and internal necrosis of bay laurel associated with severe infestations of bark or ambrosia beetles, the aims of this study were to: i) characterize the fungal species associated with stem blight and internal necrosis of bay laurel plants; ii) assess their potential pathogenicity on healthy bay laurel plants; iii) identify the insect species co-occurring with these fungal phytopathogens in the diseased plants.

## ﻿Materials and methods

### ﻿Sampling, fungal isolation and beetle identification

Field samplings were carried out in June 2021 at nurseries located in Giarre (37°41.81'N, 15°11.52'E) and Mascali (37°44.85'N, 15°12.25'E) (Catania, Italy). Stem sections from 25 bay laurel plants exhibiting symptoms of stem blight, internal necrosis, and beetle infestations were randomly collected. Samples were then placed in sterile plastic bags and transferred to the laboratory of Plant Pathology at the Department of Agriculture, Food and Environment, University of Catania, for fungal isolation and insect identification. In the laboratory, 180 small wood fragments were collected from the discoloured inner woody tissues surrounding the outer portions of the beetle-bored galleries, excluding the interior of the galleries, which are typically inhabited by beetle-specific fungal symbionts. This approach was taken to focus on isolating the fungal species responsible for causing the disease. Each fragment was then divided into subsections (5 × 5 × 5 mm), with priority given to areas where the necrotic lesions were actively progressing (terminal discolored part). The subsections were sterilized in a 1.2% sodium hypochlorite solution for 60 s, rinsed once in sterile distilled water for 60 s, and air-dried in a laminar-flow hood on sterile paper. The surface-sterilized wood pieces were placed onto potato dextrose agar (PDA, Lickson, Vicari, Italy), supplemented with 100 mg L^-1^ of streptomycin sulphate (Sigma-Aldrich, St. Louis, MO, USA) to prevent bacterial growth, and then incubated at 25 ± 1 °C for ten days. The isolation frequency was calculated according to the formula: F = (N_f_/N_tot_) × 100, where F is the frequency of putative fungal pathogen, N_f_ is the number of wood subfragments from which a fungal colony of interest emerged, and N_tot_ is the total number of wood subfragments cultured on PDA. Colonies of interest were subcultured on PDA plates to obtain pure cultures for macro- and microscopic observations and DNA extraction.

Twelve representative colonies of the most frequently recovered fungus were selected to obtain single-spore isolates and were stored in the collection of the Department of Agriculture, Food and Environment, Plant Pathology Section, University of Catania, for further analyses. Several beetles were extracted from the infested laurel stem sections, individually placed into sterile vials, and transferred to the Section of Applied Entomology of the same Department for beetle species identification. All adult beetles were then observed under a stereomicroscope for morphological identification, following the keys provided by Rabaglia et al. (2006).

### ﻿Morphological characterization of fungal species

Three representative isolates (ALF2, ALF6, and ALF11) were selected for the description of pure cultures and morphological characterization. Cultures were grown on potato dextrose agar (PDA) and on cornmeal agar (Sigma, St Louis Missouri), amended with 2% (w/v) D(+)-glucose-monohydrate (CMD) at room temperature and ambient light. Colony features, such as texture, obverse and reverse colour, margin, and zonation were recorded. To examine sporulating structures, microscope slides were prepared in 3% KOH and observed at 100 × magnifications using a Zeiss Axio Imager.A1 compound microscope (Oberkochen, Germany) equipped with a Zeiss Axiocam 506 colour digital camera. Specifically, the length and width of conidia, as well as the length and width of conidiogenous cells (phialides) at their base, were measured, and the length-to-width ratio of conidia was calculated. Measurements of conidia are reported as maxima and minima in parentheses and the mean plus and minus the standard deviation of several measurements given in parentheses.

Holotype isolate (ALF11) was used to assess the effect of the temperature on mycelial growth rate. The fungal isolate was grown on PDA at 25 ± 1 °C for 14 days in the dark. Mycelial plugs, 5 mm in diameter, were obtained from the margins of actively growing colonies using a sterile cork borer and placed in the center of Petri plates, containing PDA amended with 100 mg L^-1^ of streptomycin sulphate. The plates were incubated at 5, 10, 15, 20, 25, 30, 35 and 40 ± 1 °C for 14 days in darkness. Five Petri plates were used as replicates for each temperature. Two perpendicular diameters were measured using a scale ruler at 7 and 14 days post-inoculation.

The isolates used in this study are maintained in the culture collection of the Department of Agriculture, Food, and Environment, University of Catania. Moreover, three representative isolates (AL2, ALF6 and ALF11) were deposited at the Westerdijk Fungal Biodiversity Institute (CBS), Utrecht, the Netherlands. Dried sporulating cultures were deposited as voucher specimens in the fungarium of the Department of Botany and Biodiversity Research, University of Vienna (WU-MYC).

### ﻿DNA extraction, PCR amplification and sequencing

Collected isolates were grown on PDA for 14 days for the genomic DNA extraction. Mycelium was scraped off and processed according to the manufacturer’s protocol using the Wizard Genomic DNA Purification Kit^®^ (Promega Corporation, Madison, WI, USA). DNA samples were stored at 4 °C until use. The following loci were amplified and sequenced: the complete internally transcribed spacer region (ITS1-5.8S-ITS2) rDNA gene with primers ITS5 and ITS4 (White et al. 1990); an approximately 0.9 kb fragment of the large-subunit (LSU) ribosomal RNA gene with primers LR0R (Moncalvo et al. 1995) and LR5 (Vilgalys and Hester 1990); a ca. 0.9 kb fragment of the partial alpha-actin (*act1*) gene with primers Act-1 and Act-5ra (Voigt and Wöstemeyer 2000); a ca. 0.9 kb fragment of the DNA-directed RNA polymerase II second largest subunit (*rpb2*) gene with primers RPB2-5F2 (Sung et al. 2007) and fRPB2-7cr (Liu et al. 1999); a ca. 0.8 kb fragment of the translation elongation factor 1-alpha (*tef1*) gene with primers TEF1_INTF (Jaklitsch 2009) and TEF1-LLErev (Jaklitsch et al. 2005); a ca. 0.5 kb fragment of the *tef1* gene with primers EF-688F and EF-1251R (Carbone and Kohn 1999); a ca. 0.85 kb fragment of the beta tubulin (*tub2*) gene with primers tub-intF (5’-AACAAGTAYGTYCCTCGCGCCGT-3’) and T22D (Voglmayr et al. 2019) and a ca. 0.4 kb fragment of the same gene with primers bt2a and bt2b (Glass and Donaldson 1995).

The PCR amplification products were estimated visually by electrophoresis on 1% agarose gels and subsequently purified using an enzymatic PCR cleanup (Werle et al. 1994), as described in Voglmayr and Jaklitsch (2008). PCR products were sequenced in both directions by Macrogen Inc. (Seoul, South Korea) or at the Department of Botany and Biodiversity Research, University of Vienna, using the ABI PRISM Big Dye Terminator Cycle Sequencing Ready Reaction Kit v. 3.1 (Applied Biosystems, Warrington, UK) and the same primers as in PCR. Sequencing was performed on an automated DNA sequencer (3730xl Genetic Analyser, Applied Biosystems). The DNA sequences generated were assembled with Lasergene SeqMan Pro (DNASTAR, Madison, WI, USA) and deposited in GenBank (https://www.ncbi.nlm.nih.gov/) (Table 1).

**Table 1.** Information on fungal isolates deposited in GenBank and used in the phylogenetic analyses.

Taxon	Isolates^a^	Status^b^	Substrate/Host	GenBank accession numbers^c^					
				ITS	LSU	*act1*	*rpb2*	*tef1*	*tub2*
*Annulusmagnustriseptatus*	CBS 128831		decayed driftwood of *Alnusglutinosa*		GQ996540		JQ429258		
*Ascitendusaustriascus*	CBS 131685		decayed driftwood of *Alnusglutinosa*		GQ996539		JQ429257		
*Myrmecridiummontsegurinum*	JF 13180	HT	submerged wood of *Fraxinusexcelsior*	KT991674	KT991664		KT991654		
*Phialemoniopsishipposidericola*	KUMCC 21-0778	HT	*Hipposideroslarvatus*	ON426882	OP363279	OQ930298			OR025957
*Phialemoniopsishipposidericola*	KUMCC 21-0779		*Hipposideroslarvatus*	ON426886	OP363283	OQ930299			OR025958
*Phialemoniopsisxishuangbannaensis*	KUMCC 21-0774	HT	*Hipposideroslarvatus*	ON426881	OP363278	OQ930300			OR025959
*Phialemoniopsisxishuangbannaensis*	KUMCC 21-0775		*Hipposideroslarvatus*	ON426884	OP363281	OQ930301			OR025960
*Phialemoniopsisxishuangbannaensis*	KUMCC 21-0776		*Hipposideroslarvatus*	ON426885	OP363282	OQ930302			OR025961
*Phialemoniopsisxishuangbannaensis*	KUMCC 21-0777		*Hipposideroslarvatus*	ON426883	OP363280	OQ930303			OR025962
*Thyridiumcornearis*	CBS 131711	HT	human corneal fluid	KJ573445	KJ573450	HE599252		LC382144	HE599301
*Thyridiumcornearis*	UTHSC 06-1465		shin aspirate	HE599285	HE599270	HE599253			HE599302
*Thyridiumcurvatum*	CBS 490.82	HT	skin lesion	AB278180	AB189156	HE599258		LC382142	HE599307
*Thyridiumcurvatum*	UTHSC R-3447		human eye	HE599291	FR745927	HE599259			HE599308
*Thyridiumendophyticum*	ACCC 38979		lower stem of *Luffacylindrica* (endophyte)	KT799556	KT799556	KT799553			KT799562
*Thyridiumendophyticum*	ACCC 38980	HT	lower stem of *Luffacylindrica* (endophyte)	KT799557	KT799560	KT799554			KT799563
*Thyridiumflavostromatum*	MAFF 247509	HT	dead twigs of *Phyllostachyspubescens*	LC655959	LC655963	LC655979	LC655967	LC655971	LC655975
*Thyridiumhongkongense*	HKU39	HT	the right forearm nodule biopsy of a human	KJ573442	KJ573447	KJ573452			KJ573457
*Thyridiumlimonesiae*	CBS 146752	HT	Skin nodule	MW050977	MW050976	MW349126			MW048608
*Thyridiumoculorum*	CBS 110031	HT	human keratitis	KJ573444	KJ573449	HE599247		LC382145	HE599296
*Thyridiumoculorum*	UTHSC 05-2527		peritoneal dialysis catheter	HE599281	HE599266	HE599249			HE599298
*Thyridiumpluriloculosum*	CBS 131712	HT	human toe nail	HE599286	HE599271	HE599254		LC382141	HE599303
*Thyridiumpluriloculosum*	MAFF 247508		dead wood of *Betulamaximowicziana*	LC655960	LC655964	LC655980	LC655968	LC655972	LC655976
*Thyridiumpluriloculosum*	UTHSC 09-3589		synovial fluid	HE599287	HE599272	HE599255			HE599304
*Thyridiumpunctulatum*	MAFF 239669		dead culms of *Phyllostachyspubescens*	LC655961	LC655965	LC655981	LC655969	LC655973	LC655977
*Thyridiumpunctulatum*	MAFF 247510	ET	dead twigs of Phyllostachysnigravar.nigra	LC655962	LC655966	LC655982	LC655970	LC655974	LC655978
*Thyridiumvestitum*	CBS 125582		dead branches of *Pyruscommunis*	MH863721	MH875182				
*Thyridiumvestitum*	CBS 113027		dead bark of *Acer pseudoplatanus*		AY544671		DQ470890	DQ471058	
*Thyridiumlauri*	**ALF1**		*Laurusnobilis*	**PP907005**	**PP907017**	**PP909726**	**PP909730**	**PP909742**	**PP909754**
*T.lauri*	**ALF2 (CBS 151896)**		*Laurusnobilis*	**PP907006**	**PP907018**		**PP909731**	**PP909743**	**PP909755**
*T.lauri*	**ALF3**		*Laurusnobilis*	**PP907007**	**PP907019**		**PP909732**	**PP909744**	**PP909756**
*T.lauri*	**ALF4**		*Laurusnobilis*	**PP907008**	**PP907020**		**PP909733**	**PP909745**	**PP909757**
*T.lauri*	**ALF5**		*Laurusnobilis*	**PP907009**	**PP907021**		**PP909734**	**PP909746**	**PP909758**
*T.lauri*	**ALF6 (CBS 151897**)		*Laurusnobilis*	**PP907010**	**PP907022**	**PP909727**	**PP909735**	**PP909747**	**PP909759**
*T.lauri*	**ALF8**		*Laurusnobilis*	**PP907011**	**PP907023**		**PP909736**	**PP909748**	**PP909760**
*T.lauri*	**ALF10**		*Laurusnobilis*	**PP907012**	**PP907024**		**PP909737**	**PP909749**	**PP909761**
*T.lauri*	**ALF11 (CBS 151898)**	HT	*Laurusnobilis*	**PP907013**	**PP907025**	**PP909728**	**PP909738**	**PP909750**	**PP909762**
*T.lauri*	**ALF14**		*Laurusnobilis*	**PP907014**	**PP907026**		**PP909739**	**PP909751**	**PP909763**
*T.lauri*	**ALF17**		*Laurusnobilis*	**PP907015**	**PP907027**		**PP909740**	**PP909752**	**PP909764**
*T.lauri*	**ALF19**		*Laurusnobilis*	**PP907016**	**PP907028**	**PP909729**	**PP909741**	**PP909753**	**PP909765**

^a^ Strains and sequences generated in this study are shown in bold. ^b^ ET = epitype; HT = holotype. ^c^ ITS, internal transcribed spacer; LSU, large-subunit ribosomal RNA gene; *act1*, the partial alpha-actin gene; *rpb2*, DNA-directed RNA polymerase II second largest subunit gene; *tef1*, translation elongation factor 1-α; *tub2*, beta-tubulin.

### ﻿Phylogenetic analyses

The sequences obtained in this study were compared with NCBI GenBank nucleotide database using the standard nucleotide Basic Local Alignment Search Tool (BLAST) (https://blast.ncbi.nlm.nih.gov/Blast.cgi). All single-spore isolates (ALF1, ALF2, ALF3, ALF4, ALF5, ALF6, ALF8, ALF10, ALF11, ALF14, ALF17, ALF19) were used for phylogenetic analyses within a combined matrix of ITS rDNA, LSU, *act*, *rpb2*, *tef1*, and *tub2* sequences. The newly generated sequences of each genomic region were aligned to reference sequences of *Thyridium* downloaded from GenBank, with two species from the Annulatascales and one species of *Myrmecridium* (Myrmecridiales) selected as the outgroup. For *Thyridium*, 10 accepted species and two recently described species of *Phialemoniopsis* for which sequences were available were included in the matrix, including ex-epitype and ex-holotype strains. The GenBank accession numbers of the sequences used in these analyses are given in Table 1.

Sequence alignments for phylogenetic analyses were produced with the server version of MAFFT (https://mafft.cbrc.jp/alignment/server/) and checked and refined using BioEdit Sequence alignment Editor 7.7.1.0 (Hall 1999). All six loci (ITS, LSU, *act1*, *rpb2*, *tef1*, *tub2*) were concatenated to a combined matrix using Phyutility v. 2.2 (Smith and Dunn 2008). The combined data matrix for phylogenetic analyses contained 4257 characters (528 nucleotides of ITS, 816 nucleotides of LSU, 839 nucleotides of *act1*, 879 nucleotides of *rpb2*, 785 nucleotides of *tef1*, and 410 nucleotides of *tub2*). Maximum likelihood (ML) analyses were performed with RAxML (Stamatakis 2006), as implemented in raxmlGUI 2.0 (Silvestro and Michalak 2012), using the ML + rapid bootstrap setting and the GTRGAMMA+I substitution model which was selected as the most appropriate model by Modeltest. The matrix was partitioned for the different gene regions, and bootstrap analyses were done with 1,000 bootstrap replicates. For evaluation and interpretation of bootstrap support, values between 70% and 90% were considered moderate, above 90% as high, and 100% as the maximum. Maximum parsimony (MP) bootstrap analyses were performed with Phylogenetic Analyses Using Parsimony (PAUP) v. 4.0a169 (Swofford 2002). A total of 1,000 bootstrap replicates were implemented using five rounds of heuristic search with random sequence addition, followed by tree-bisection-reconnection (TBR) branch swapping. The MULTREES option was enabled, the steepest descent option was disabled, the COLLAPSE command was set to MINBRLEN, and each replicate was limited to 1 million rearrangements. All molecular characters were treated as unordered and assigned equal weight, with gaps considered as missing data. The COLLAPSE command was set to MINBRLEN.

### ﻿Pathogenicity test

To assess the pathogenicity of *T.lauri*, two-year-old potted bay laurel plants grown under controlled growth chamber conditions were used. The holotype isolate (ALF11) was grown on PDA amended with 100 mg L^-1^ of streptomycin sulphate and incubated at 25 ± 1 °C for 30 days. The stems were surfaced disinfected with a 70% ethanol solution, and the bark was gently scraped using a sterile blade. The mycelial plug was then placed upside down onto the wound. Wounds were sealed with Parafilm to prevent desiccation. All inoculated plants were moved to a growth chamber with a 12 h photoperiod and maintained at 25 ± 1 °C. The plants were regularly watered and monitored monthly for symptom development. A total of nine plants were inoculated with *T.lauri* ALF11, while the control group consisted of an equal number of plants inoculated with sterile PDA. After four months, the phloem tissue was peeled back, and the lesion length extending both upward and downward from each inoculation site was measured. The mean and standard deviation were calculated. Pieces of necrotic tissue were cultured as previously described to fulfill the Koch’s postulates, and the frequency of *Thyridium*-like colonies was determined.

## ﻿Results

### ﻿Sampling, fungal isolation and beetle identification

**Figure 1.**
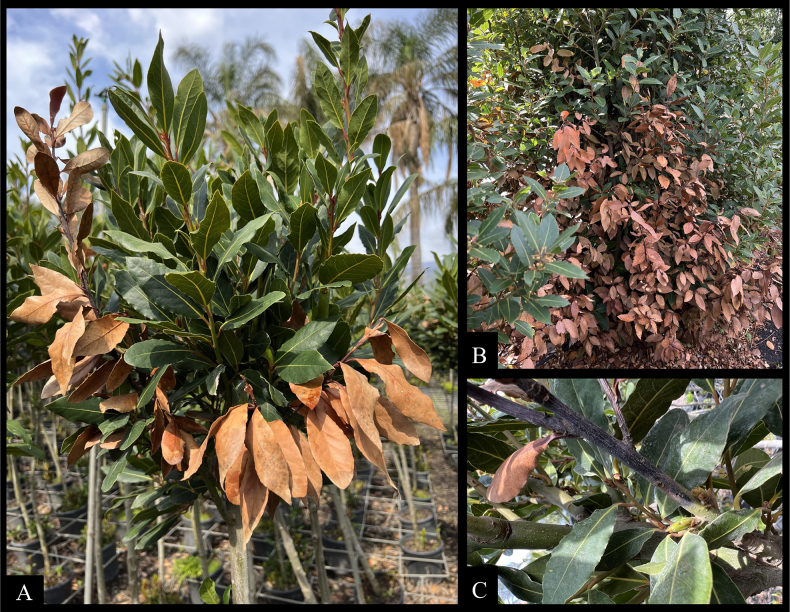
Symptoms of stem blight on bay laurel plants in nurseries located in Catania province **A** stem blight of twigs on a four-year-old tree, typically observed on a section of the canopy **B** stem blight of the lower part of plant **C** stem discoloration progressing from the tips to the lower part of the twig (red arrow).

**Figure 2.**
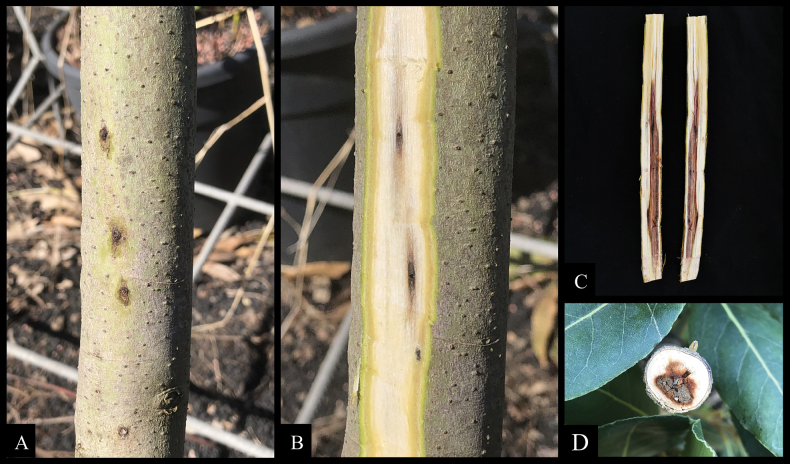
Details of symptoms on bay laurel trees **A, B** bark necrosis surrounding the small circular entry holes of *Xylosandruscompactus***C, D** longitudinal and cross-section of twigs showing internal necrosis reaching the pith.

In the surveyed nurseries, bay laurel plants consistently showed symptoms of stem blight. The initial external symptoms observed included blight of the terminal leaves, beginning at the tips and progressing downward along the twigs. The leaves became dry as though suffering from lack of water and remained attached to twigs for several months (Fig. 1). In the most severe cases, the entire tree died. Symptomatic plants consistently exhibited bark necrosis surrounding small, circular entry holes bored by beetles, with diameters ranging from approximately 0.7 to 0.9 mm (Fig. 2a). Upon removing the bark from symptomatic stems or twigs with beetle entry holes, black to brown streaks of discoloration were observed in the sapwood, extending from the entry points into the surrounding wood (Fig. 2b–d). Longitudinal sections of the stem revealed discoloration extending upwards and downwards from the beetle galleries. Disease incidence, based on the number of plants showing symptoms of stem blight associated with ambrosia beetle attack in the nurseries where the disease was first observed (Mascali), ranged from approximately 30% to 40% of the 3,000 total plants. Two types of fungal colonies were consistently recovered from symptomatic tissue, with isolation frequencies ranging from 60 to 95% for *Thyridium*-like colonies and from 5 to 40% for *Ambrosiella*-like colonies. More than 300 adult beetle females were collected from diseased samples all of which were morphologically identified as *X.compactus*, the sole beetle species present in the sampled woody material.

### ﻿Phylogenetic analyses and fungal species identification

**Figure 3.**
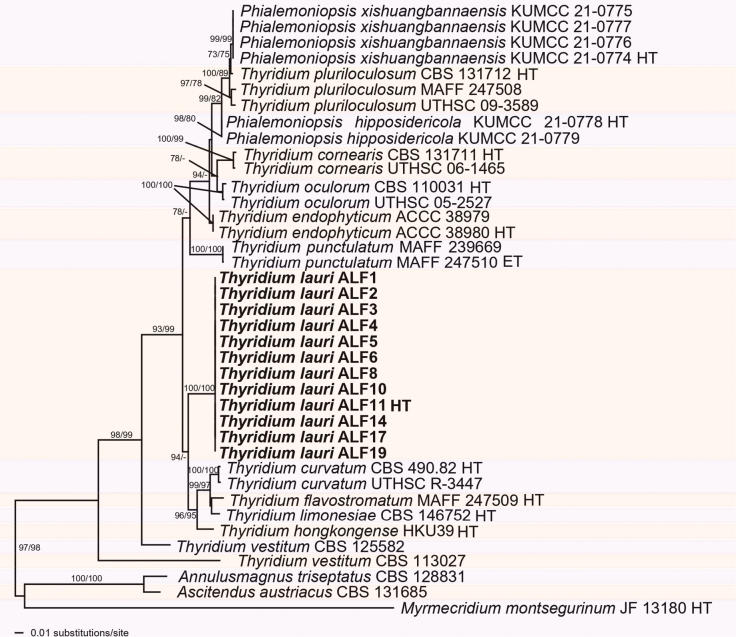
Phylogram of the best ML tree (-lnL = 13574.613578) revealed by RAxML from an analysis of the combined ITS-LSU-*act1*-*rpb2*-*tef1*-*tub2* matrix of *Thyridium*, showing the phylogenetic position of the new species from diseased *Laurusnobilis* (bold), with *Annulusmagnustriseptatus*, *Ascitendusaustriacus* and *Myrmecridiummontsegurinum* selected as outgroup to root the tree. Maximum Likelihood (ML) and Maximum Parsimony (MP) bootstrap support above 70% are given at first and second position, respectively, above or below the branches. ET = epitype; HT = holotype.

Of the 4257 characters of the combined matrix used for phylogenetic analyses, 593 were parsimony informative (44 from ITS, 68 from LSU, 102 from *act1*, 259 from *rpb2*, 62 from *tef1*, and 51 from *tub2*), 364 were parsimony-uninformative and 3300 were constant. The ML tree (-lnL = 13574.613578) obtained by RAxML is shown in Fig. 3. Maximum Likelihood analyses resulted in a tree topology similar to that revealed by MP bootstrap analysis. The monophyly of the genus *Thyridium* was strongly supported, with 97% support in the ML analysis and 98% in the MP analysis, as was the monophyly of most *Thyridium* species included. However, the two non-type strains of *T.vestitum* (CBS 113027, CBS 125582) did not form a monophyletic clade, indicating that at least one of the two isolates is misidentified. The twelve isolates of this study formed a distinct monophyletic clade within *Thyridium*, representing a new species with maximum support. This clade was resolved as a sister group to a highly supported clade (96% ML, 95% MP) containing *T.curvatum*, *T.flavostromatum*, *T.hongkongense*, and *T.limonesiae*. While most terminal and basal nodes received high to maximum support, intermediate nodes were generally poorly supported.

### ﻿Pathogenicity test

**Figure 4.**
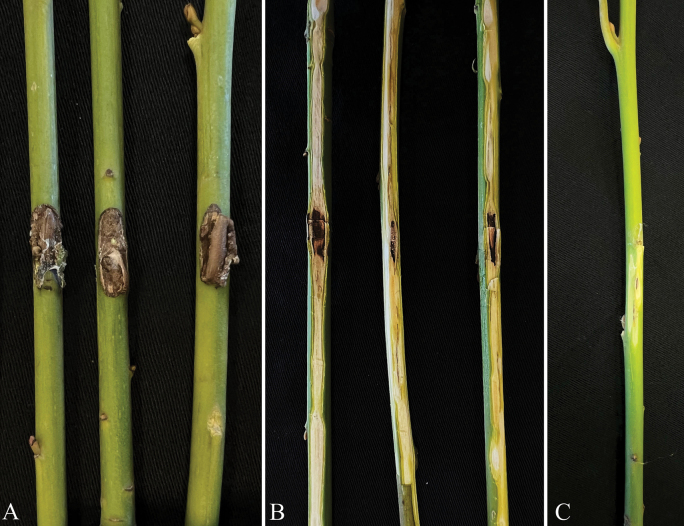
Pathogenicity test on bay laurel **A** stems inoculated with *Thyridiumlauri* isolate ALF11, showing symptoms of external lesion **B** internal necrosis with brownish to blackish streaking in the sapwood **C** control plant inoculated with sterile PDA.

The pathogenicity test confirmed that the new *Thyridium* species is pathogenic to bay laurel, causing internal necrosis and stem blight of all inoculated plants. The initial visible external symptoms included brown to black necrotic lesions at the inoculation site (Fig. 4), followed by the development of stem blight within four months. Internally, plants exhibited discoloration, characterized by brownish to blackish streaking in the sapwood, with a mean lesion length of 16.6 ± 7.1 cm. In addition, some inoculated and control plants exhibited brown exudates, which over time became crusty and adherent to the bark as a response to mechanical damage. Control plants displayed no internal symptoms, aside from minor wound oxidation. *Thyridium* was consistently re-isolated from necrotic tissue but was absent in the control treatments. The re-isolation frequency was 94%, and the emerging fungal colonies matched the originally inoculated *Thyridium* isolates based on morphological observations of the colony and conidia. Therefore, Koch’s postulates were fulfilled.

### ﻿Growth rate experiments

**Figure 5.**
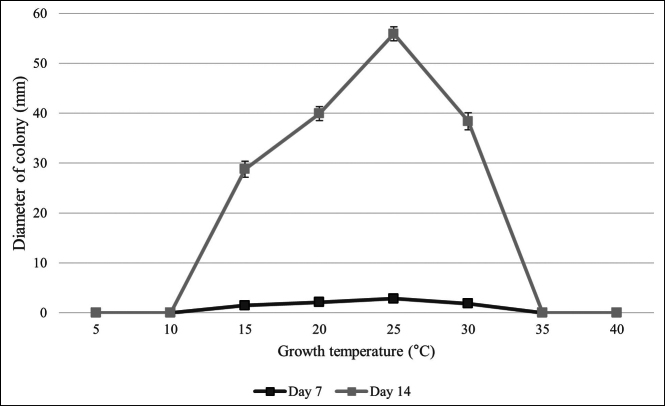
Effect of temperature on mycelial growth rate of *Thyridiumlauri* ALF11 isolated from bay laurel. Data are means of five replicated Petri plates with bars indicating the standard error (SE).

The growth rate experiments of *T.lauri* isolate ALF11 are shown in Fig. 5. The fungus grew slowly at 15 °C (29 mm diameter after 14 days). No mycelial growth was observed at 0, 5, 10, 35, and 40 °C while optimal growth occurred within the temperature range of 20 to 30 °C.

### ﻿Taxonomy

#### ﻿ Thyridium lauri

Taxon classificationFungiThyridialesThyridiaceae

Voglmayr, D. Aiello & G.R. Leonardi sp. nov.

019233DB-753C-51B5-AAF4-DF771A1C30B4

MycoBank No: 854792

Figs 6 , 7

##### Etymology.

Referring to its host, *Laurusnobilis*.

##### Type.

Italy • Sicily, Catania province, plant nursery located in Mascali, 37°44.85'N, 15°12.25'E, isolated from diseased corticated twigs of *Laurusnobilis*, 23 June 2021, G. Polizzi (holotype WU-MYC 0052725 dried culture; ex-holotype culture ALF11 = CBS 151898).

##### Description.

On PDA reaching 54–56 mm diameter after 21 days, first pale creamy to yellowish, cottony in the centre, with dense whitish aerial mycelium after 14 days, after 1 month colonies ochraceous to umber brown in the centre, with distinct concentric lighter and darker zones, aerial mycelium white or grayish in the centre, sparse or lacking towards the margins, forming dry effused patches of branched aerial conidiophores interspersed with spot-like to effused patches of densely branched conidiophores immersed in white to cream slimy conidial masses; reverse dark umber brown in the centre, with lighter and darker brown concentric zones towards the margins. On CMD reaching 59–62 mm diameter after 21 days, colonies first whitish, from the centre becoming greenish brown with age, finally blackish brown in the centre, aerial mycelium sparse to absent, with scant erect branched conidiophores and strands of aggregated radial hyphae on the agar surface. Sporodochia and pycnidia not observed. Conidiation of three types, all of (sub)hyaline, thin-walled cells: (1) on tufts of aerial, several times loosely branched conidiophores, with straight elongate hyphal conidiophore cells and cylindrical to narrowly-ampulliform terminal or lateral conidiogenous cells (phialides) of variable length up to 50 µm long, producing conidia terminally; (2) on densely branched, interwoven-aggregated conidiophores, with knobby to sinuous conidiophore cells and terminal or lateral conidiogenous cells (polyphialides) of variable, irregular flexuous-knobby shape, 4–18 × 1.2–3.6 µm (n = 155), forming conidia terminally and/or laterally; and (3) on straight to bent adelophialides formed singly on hyphae at more or less right angles, 1–13 × 0.7–1.8 μm (n = 56), with a tiny pericline apical thickening, but without visible collarettes. Conidia hyaline to subhyaline, thin-walled, of two types: (1) subglobose to broadly ellipsoid, uni- to irregularly multiguttulate, commonly aggregated in spot-like to effuse slimy masses, produced on the branched conidiophore type (2), (2.2–)3.0–3.8(–4.8) × (2.0–)2.3–3.0(–3.7) μm, l/w = (1.0–)1.2–1.5(–1.9) (n = 211); (2) elongate, ellipsoid to allantoid, mostly biguttulate with a guttule near each end, mainly borne from adelophialides but also on conidiophore type (1), (2.3–)3.0–4.0(–4.9) × (1–)1.3–2.0(–2.8) μm, l/w = (1.2–)1.8–2.8(–4.0) (n = 169). Subglobose to broadly ellipsoid conidia mainly produced in masses on PDA in older parts of the colony; elongate to allantoid conidia commonly observed on CMD, more rarely on PDA, produced within 3–4 days mainly in the actively growing younger parts of the colony. On inoculation plugs of PDA cultures placed on CMD yeast-like cells observed 3 to 4 days after inoculation, developing by budding at one or two ends from swollen subglobose conidia. Sexual morph unknown.

**Figure 6.**
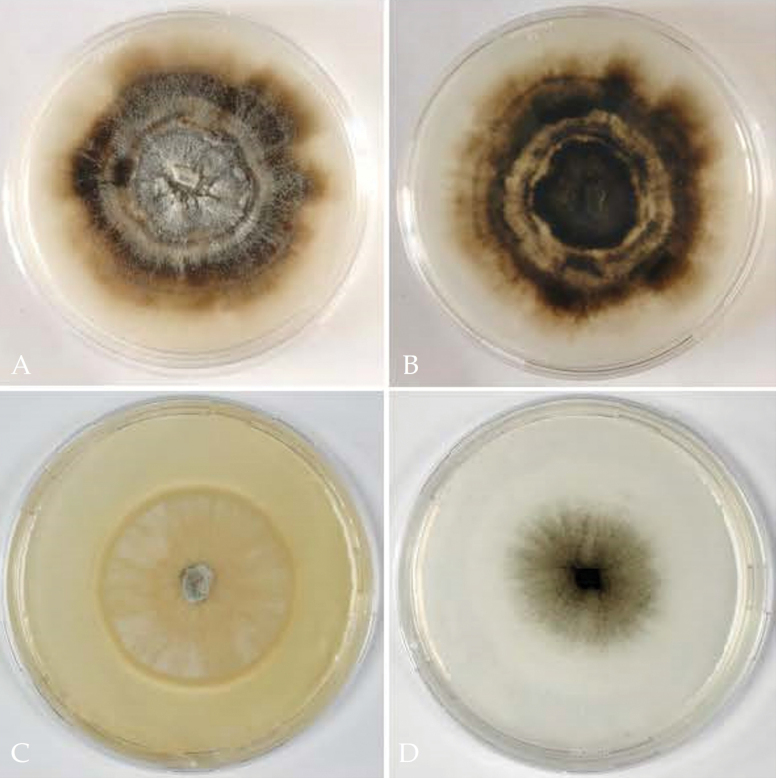
Colonies of *Thyridiumlauri* (ALF 11, ex-holotype culture) **A** on PDA (1 month, 22 °C) **B** on PDA (1 month, 22 °C), reverse **C** on ½ PDA (21 days, 22 °C) **D** on CMD (21 days, 22 °C).

**Figure 7.**
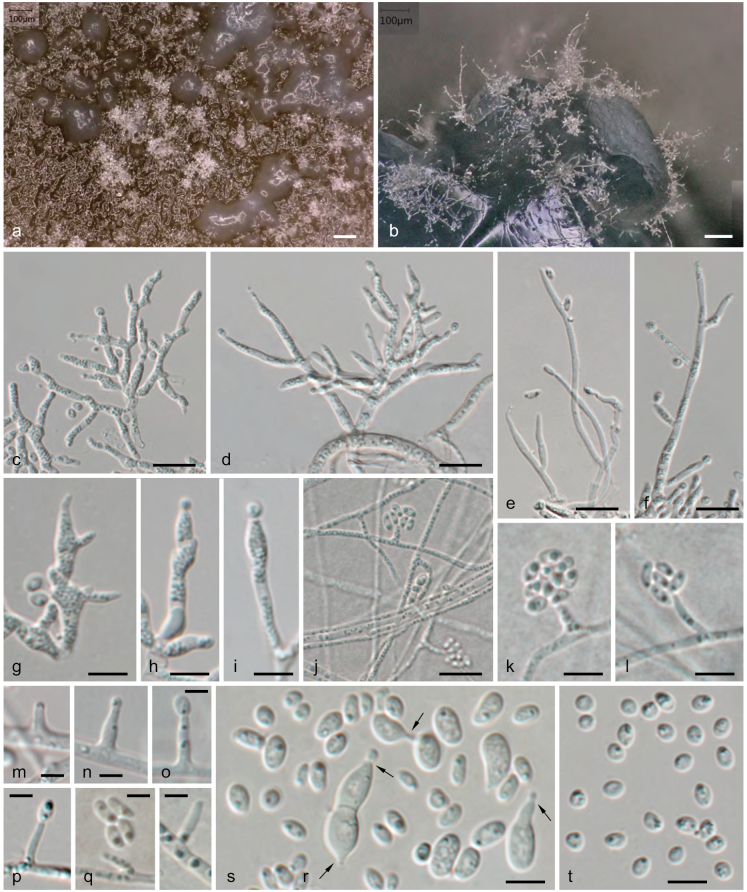
Morphology of *Thyridiumlauri* (**a–c, e–h, t.** ALF6; **d, i, j, k, m–o, r, s.** ALF11; **l, p, q.** ALF2) **a** tufts of aerial conidiophore type 1 interspersed with patches of conidiophore type 2 submerged in slimy conidal masses (PDA, 1 month) **b** inoculation plug of PDA culture on CMD showing branched aerial conidiophore type 1 (11 days) **c** densely branched conidiophore type 2, showing flexuous-knobby conidiophore cells and irregular terminal and lateral conidiogenous cells (PDA, 1 month) **d–f** loosely branched conidiophore type 1 showing straight terminal and lateral conidiophore and conidiogenous cells (PDA, 1 month) **g, h** irregular conidiogenous cells from conidiophore type 2, showing terminal and lateral conidiation (PDA, 1 month) **i** ampulliform conidiogenous cell from conidiophore type 1 (CMD, 11 days) **j** surface mycelium with hyphal strand and adelophialides with ellipsoid-allantoid conidia (CMD, 11 days) **k–r** adelophialides on hyphae with ellipsoid-allantoid conidia (**k, l, p–r**CMD, 11 days; **m–o**PDA, 3 days) **s** swollen conidia showing yeast-like budding (arrows; PDA plug on CMD, 3 days) **t** subglobose conidia from conidiophore type 2 (PDA, 1 month). All from pure cultures grown at 22 °C. Scale bars: 100 μm (**a, b**); 10 μm (**c–f, j**); 5 μm (**g–i, k, l, s, t**); 3 μm (**m–r**).

##### Additional specimens examined.

Italy • Sicily, Catania province, plant nursery located in Giarre, 37°41.81'N, 15°11.52'E, isolated from twig necrosis of *Laurusnobilis*, 10 June 2021, G. Polizzi (WU-MYC 0052726, dried culture ALF2; CBS 151896); • Catania province, plant nursery located in Giarre, 37°41.81'N, 15°11.52'E, isolated from twig necrosis of *Laurusnobilis*, 10 June 2021, G. Polizzi (WU-MYC 0052727, dried culture ALF6; CBS 151897).

Based on the results of molecular phylogenies (Fig. 3) and the synonymy of *Phialemoniopsis* with *Thyridium* two recently described *Phialemoniopsis* species are here combined in *Thyridium*.

#### ﻿ Thyridium hipposidericola

Taxon classificationFungiThyridialesThyridiaceae

(Karun., Tibpromma & X.F. Liu) Voglmayr comb. nov.

B1789168-8ED5-5318-8346-39FAC2D46D3F

MycoBank No: 854793

Phialemoniopsis hipposidericola Karun., Tibpromma & X.F. Liu, in Liu et al. (2023). Basionym.

#### ﻿ Thyridium xishuangbannaense

Taxon classificationFungiThyridialesThyridiaceae

(Karun., Tibpromma & X.F. Liu) Voglmayr comb. nov.

6BB79D7B-CB54-519E-A461-98D975B78B98

MycoBank No: 854794

Phialemoniopsis xishuangbannaensis Karun., Tibpromma & X.F. Liu, in Liu et al. (2023). Basionym.

## ﻿Discussion

This study revealed stem blight of bay laurel in Southern Italy as being associated with a new fungal species within the genus *Thyridium* (Sordariomycetes, Thyridiaceae). The isolates obtained from symptomatic tissues were identified based on the morphological characteristics and molecular phylogenetic analyses of the ITS, LSU, *act1*, *rpb2*, *tef1*, and *tub2* gene regions. Recently, Sugita and Tanaka (2022) provided a phylogenetic treatise on the genus *Thyridium*, which included the same six loci sequenced in our study. Molecular evidence and morphological similarities in the asexual morph revealed that the anamorph genus *Phialemoniopsis* is congeneric with *Thyridium*, leading to the reclassification of eight *Phialemoniopsis* species into *Thyridium* (Sugita and Tanaka 2022). Currently, molecular data for 10 *Thyridium* species and 2 *Phialemoniopsis* species originating from different substrates, including humans, animals, and plants, are available in GenBank. According to Index Fungorum (https://www.indexfungorum.org/Names/Names.asp; Accessed April 19, 2024), a total of 65 *Thyridium* species have been described, of which 44 are currently recognised within the genus *Thyridium*. However, sequence data are not yet available for the majority of these species, with 34 lacking molecular information. Two species of *Phialemoniopsis*, *P.hipposidericola* and *P.xishuangbannaensis*, recently described from bats in Southern China (Liu et al. 2023), are also classified within *Thyridum* based on molecular phylogenies (Fig. 3) and are therefore combined in *Thyridium* in this study.

In this study, the sequenced isolates formed a clade that is significantly distinct from all known *Thyridium* species with available sequence data, and are therefore described as a new species, *T.lauri*. Our isolates are phylogenetically close to *T.curvatum*, *T.flavostromatum*, *T.hongkongense*, and *T.limonesiae*, which were recovered from parts of human body (Perdomo et al. 2013; Tsang et al. 2014) or associated with human infections (Perdomo et al. 2013; Alvarez Martinez et al. 2021). *Thyridiumlauri* shares similar morphological characteristics with other *Thyridium* species, with only minimal distinct traits observed. Nevertheless, the newly proposed species does not produce pycnidium-like conidiomata or chlamydospores unlike many phialemoniopsis-like asexual morphs of *Thyridium*. Similarly, no pycnidium-like conidiomata or chlamydospores have been observed in *T.curvatum* (Perdomo et al. 2013), *T.hongkongense* (Tsang et al. 2014), and *T.limonesiae* (Alvarez Martinez et al. 2021). *Thyridiumlauri* is characterised by the production of subglobose conidia aggregated in slimy masses and of yeast-like cells developing by budding from the same type of conidia. Moreover, it can be morphologically distinguished from the other species of the sister clade. The new species is distinguished from *T.hongkongense* by its larger elongate, ellipsoid to allantoid conidia (2.3–4.9 × 1.3–2.8 μm), while conidia of *T.hongkongense* are small, cylindrical, oval or rod- shaped, and slightly curved (2–3 × 1–2 μm) (Tsang et al. 2014). Similarly, *T.limonesiae* produces shorter cylindrical or oval conidia (2.3–4.9 × 1.4–2 μm) (Alvarez Martinez et al. 2021). Although *T.lauri* appears quite similar to *T.curvatum* in producing two types of conidia, *T.curvatum* is distinguished by its longer allantoid conidia (4–6 × 1–2 μm), shorter ellipsoid to ovoid conidia (1–2 × 0.5–1), and the presence of sporodochial conidiomata which were not found in *T.lauri* or *T.flavostromatum* (Perdomo et al. 2013). Within this sister group, *T.flavostromatum* produces the longest ellipsoid to allantoid conidia (2–7 × 1–2.5 μm) (Sugita and Tanaka 2022), characterised by a slightly apiculate base. Unlike *T.lauri*, *T.flavostromatum* produces solitary clamydospores.

In addition to the morphological characteristics, *T.lauri* can be distinguished from other species by its notable ecological traits. The new species was isolated from two different locations in Sicily, from the same host, and was confirmed to be pathogenic to bay laurel, as demonstrated by our pathogenicity tests. Notably, several anamorphic *Thyridium* species have been reported as opportunistic human pathogens, including *T.curvatum*, *T.hongkongense*, *T.limonesiae*, *T.oculorum*, *T.pluriloculosum* (Gams and McGinnis 1983; Rivero et al. 2009; Desoubeaux et al. 2014; Tsang et al. 2014; Alvarez Martinez et al. 2021; Patolia and Bansal 2021). These species have also been isolated from various environments, including soil samples (*T.curvatum*) (Perdomo et al. 2013), diesel fuel tank (*T.curvatum*) (Varaljay et al. 2019), seawater samples (*T.pluriloculosum*) (Taha et al. 2021), and marine sediment (*T.oculorum*) (Mahanty et al. 2019). A few *Thyridium* species reported as human pathogens have also been isolated from plant material at different reproductive stages. For example, *T.pluriloculosum* originally found in human nails as an asexual fungus (Perdomo et al. 2013) and reported to cause human infections (Patolia and Bansal 2021), was later rediscovered in its sexual state on the twigs of *Betulamaximowicziana* (Sugita and Tanaka 2022). Other *Thyridium* species were isolated from plants (*T.curvatum*, *T.endophyticum*, *T.flavostromatum*, *T.punctulatum*) as non-pathogenic endophytes or saprobes (Halleen et al. 2007; Kaur et al. 2014; Su et al. 2016; Sugita and Tanaka 2022). Among these, *T.curvatum* and *T.oculorum* were also isolated from asymptomatic nursery material and young grapevines affected by Petri disease, respectively, but their importance in the grapevine decline has not yet been confirmed (Halleen et al. 2007; Ferreira et al. 2018). It is important to note that several *Thyridium* species, yet to be sequenced, have been described as sexual morphs from dead plant tissues. A review of the literature reveals that a single species, *T.nobile*, was recorded from twigs of a member of Laureaceae, *Laurusnovocanariensis* (Petrak 1929). According to the USDA Fungus-Host database (https://fungi.ars.usda.gov; Accessed April 19, 2024), *Thyridium* has been recorded neither from *Laurusnobilis* nor from other Lauraceae. We are therefore confident that our species has not yet been described. The observed morphological differences, combined with the phylogenetic analyses and ecological characteristics, support the classification of these isolates as a new species.

The pathogenicity tests on bay laurel demonstrated that *T.lauri* is responsible for causing necrotic lesions in the stems, ultimately leading to stem blight. However, *Xylosandruscompactus* may also play a significant role in the development of symptoms. Notably, during our surveys, the symptoms of stem blight observed in bay laurel were consistently associated with the presence of entry holes and galleries created by the adults of the invasive ambrosia beetle, *X.compactus*.

As reported by several authors, the tunnelling activity in the wood, which disrupts the transport of water and nutrients, along with the introduction of ambrosia fungi presumed to be pathogenic to the host trees, likely contributes to the severe stem blight or even death of the plants (Ngoan et al. 1976; Paine et al. 1997; Bosso et al. 2012; Pennacchio et al. 2012; Greco and Wright 2015). In 2011, *X.compactus* was first recorded for Europe (Garonna et al. 2012) in urban parks of the Campania region in Italy. In Sicily, this ambrosia beetle was mainly found infesting carob trees (Gugliuzzo et al. 2019a), while in other regions of Italy it has also been found infesting many other host plants, including bay laurel (Francardi et al. 2012; Bateman et al. 2016; Vannini et al. 2017). As the crucial role of arthropods in disease epidemiology has been increasingly recognized worldwide (Moyo et al. 2014; Cruz et al. 2021; Heitmann et al. 2021; Berasategui et al. 2022; Lunde et al. 2023), it cannot be excluded that the beetles may actively disseminate the fungal propagules, or alternatively provide entry points for secondary fungal infections (Brader 1964). However, specific investigations are needed to verify these hypotheses. Previous studies have demonstrated that *X.compactus* inhabits the xylem, where it introduces specific mutualistic symbiotic fungi on which it relies for nutrition. The most consistently associated symbiotic fungal species, isolated from *X.compactus* and its several hosts worldwide, were *Ambrosiellaxylebori* Brader ex Arx & Hennebert, *A.macrospora* (Francke-Grosm.) L.R. Batra, and *Fusarium* spp. (Muthappa and Venkatasubbaiah 1981; Bhat and Sreedharan 1988; Bosso et al. 2012; Bateman et al. 2016; Gugliuzzo et al. 2020, 2021). In this study, *Ambrosiella*-like colonies were isolated from symptomatic woody tissues in addition to *T.lauri*. In contrast, previous investigations did not reveal the presence of *Thyridium* involved in stem blight and internal necrosis associated with *X.compactus* (Vannini et al. 2017; Gugliuzzo et al. 2020; Benvenuti et al. 2021; Morales-Rodríguez et al. 2021; Vitale et al. 2022). However, some unidentified *Phialemonium* sp. and *Phialemoniopsis* sp. isolates were recorded in previous studies evaluating the fungal communities of *X.compactus* in the United States (Bateman et al. 2016) and Italy (Gugliuzzo et al. 2020). The ITS sequence of *Phialemonium* isolate Hulcr5398 (KU961666) (Bateman et al. 2016) matched very closely with *T.oculorum* (previously *Phialemoniopsisocularis*, OR760549, HG933293), with a percentage identity of 99.6% in the BLAST search results. Moreover, phylogenetic analyses of the *Phialemonium* sp. isolate in our multigene matrix revealed that it was nested within the *T.oculorum* clade (data not shown), confirming its placement within *Thyridium*, although phylogenetically distant from *T.lauri*. While *Phialemoniopsis* and *Phialemonium* share highly similar morphologies, they phylogenetically belong to two distinct families within Sordariomycetes, Thyridiaceae and Cephalothecaceae, respectively (Perdomo et al. 2013; Hongsanan et al. 2017; Davolos et al 2019). Isolates morphologically identified as *Phialemonium* may, in fact, represent *Phialemoniopsis*, which is now considered a synonym of *Thyridium* (Sugita and Tanaka 2022).

One interesting finding is that the ITS of *Phialemoniopsis* isolate 50 (ON520570) (Gugliuzzo et al. 2020) was identical to *Thyridium* sp. (PP683252), and it matched very closely with *Phialemoniopsis* sp. isolates V18210, V18211, V18170, V18171 (ON413722, ON413723, ON415520, ON415521) and *Acremonium* sp. isolates Hulcr5037 (KU961664) and PB_AG_03 (OQ513933), with percentage identity ranging from 99 to 99.44% in the BLAST search results. Furthermore, phylogenetic analyses of the *Phialemonium* and *Acremonium* isolates in our multigene matrix, for which ITS and three LSU sequences are available in GenBank, showed that these isolates were nested within the *T.lauri* clade with high support (data not shown). This strongly suggests that they are members of *Thyridium* and are presumably very closely related, if not the same species. The *Acremonium* isolates Hulcr5037 and PB_AG_03, isolated from *X.compactus* in the United States (Bateman et al. 2016) and from *X.germanus* in Germany (Gugliuzzo et al. 2023b), respectively, were also reported to be one of the most prevalent fungi isolated from these beetles. However, their characterisation was based solely on the ITS sequences (Bateman et al. 2016; Gugliuzzo et al. 2023b). The other *Phialemoniopsis* sequences in GenBank that are highly similar to our ITS sequences originated from an unpublished study on fungi associated with *X.compactus* in the USA (ON413722, ON413723) and China (ON415520, ON415521). These remarkable findings suggest that the association of *T.lauri* and closely related fungal species with the beetle could be more widespread and stable, rather than attributed to a recent acquisition by the beetle from another host in the same area or to a recent introduction from other countries. The misidentification of the fungi commonly isolated from *X.compactus* may be attributed to the fact that earlier studies relied solely on ITS sequences, which were the only data available at the time, and *T.lauri* had not yet been described. This study enhances our understanding of the *Thyridium*-*Xylosandrus* association, and future multi-locus analyses of *Phialemoniopsis* and *Acremonium* isolates are necessary to accurately distinguish these species.

Interestingly, we observed a yeast-like phase of *T.lauri* when subcultured on a new medium, especially on inoculation plugs during the initial growth of the colony. It is known that symbiotic fungi of ambrosia beetles have evolved in a phase that facilitates the dissemination through the insect (Cassar and Blackwell 1996; Nel et al. 2021; Mayers et al. 2022). Several studies have demonstrated that ambrosia fungi are dimorphic, exhibiting both a mycelial phase and a yeast-phase, also referred to as the sprout-cell phase (Batra 1967). Specifically, *Harringtonialauricola*, *Ceratocystislunata*, and *Raffaelapromiscua* have been reported in association with *Xyleborusaffinis*, *Xylosandruscrassiusculus*, and *Xyleborinussaxesenii*, respectively, undergoing the dimorphic transition from filamentous to yeast-like growth, which was also observed *in vitro* (Batra 1967; Nel et al. 2021; Mayers et al. 2022; Joseph et al. 2023). This finding suggests that the newly described species may have a predisposition for developing associations with arthropods. In conclusion, this study identified a new pathogenic fungal species, *Thyridiumlauri* sp. nov., responsible for causing stem blight and internal necrosis, in association with *X.compactus* infestations on bay laurel in Italy.

However, the ecological role of this fungus and its interaction with the beetle remain unclear. Further investigations are necessary to: (i) determine whether *X.compactus* plays a role in the dissemination of *T.lauri* spores by isolating *T.lauri* from *X.compactus* adults infesting bay laurel and fulfilling Leach’s postulates (Hulcr et al. 2020); and (ii) elucidate the potential symbiotic association between *T.lauri* and *X.compactus*.

## Supplementary Material

XML Treatment for Thyridium lauri

XML Treatment for Thyridium hipposidericola

XML Treatment for Thyridium xishuangbannaense

## ﻿Additional information

### Author ORCIDs

Giuseppa Rosaria Leonardi https://orcid.org/0000-0002-4676-5100

Dalia Aiello https://orcid.org/0000-0002-6018-6850

Chiara Di Pietro https://orcid.org/0009-0005-5746-8389

Antonio Gugliuzzo https://orcid.org/0000-0002-4118-2280

Giovanna Tropea Garzia https://orcid.org/0000-0002-2161-7021

Giancarlo Polizzi https://orcid.org/0000-0001-8630-2760

Hermann Voglmayr https://orcid.org/0000-0001-7666-993X

### Data availability

The data obtained in this study are available in the main text and have been publicly uploaded to NCBI GenBank and MycoBank.
